# The Tomato Wilt Fungus *Fusarium oxysporum* f. sp. *lycopersici* shares Common Ancestors with Nonpathogenic *F. oxysporum* isolated from Wild Tomatoes in the Peruvian Andes

**DOI:** 10.1264/jsme2.ME13184

**Published:** 2014-06-06

**Authors:** Keigo Inami, Takeshi Kashiwa, Masato Kawabe, Akiko Onokubo-Okabe, Nobuko Ishikawa, Enrique Rodríguez Pérez, Takuo Hozumi, Liliana Aragón Caballero, Fatima Cáceres de Baldarrago, Mauricio Jiménez Roco, Khalid A. Madadi, Tobin L. Peever, Tohru Teraoka, Motoichiro Kodama, Tsutomu Arie

**Affiliations:** 1 Graduate school of Agriculture, Tokyo University of Agriculture and Technology, Fuchu, Tokyo, 183–8509, Japan; 2 National Agricultural Research Center (NARC), Tsukuba, Ibaraki, 305–8666, Japan; 3 Department of Plant Breeding, Chapingo Autonomous University, Texcoco, CP 56230, Mexico; 4 Interdisciplinary Research Center for Environment and Rural Service, Chapingo Autonomous University, Texcoco, CP 56230, Mexico; 5 Department of Phytopathology, National Agrarian University—La Molina, Lima, Peru; 6 Department of Biology, National University of San Augustín, Arequipa, Peru; 7 Tarapacá University, a la Casilla 6-D, Arica, Chile; 8 Kabul University, Jamal Mina, Afghanistan; 9 Department of Plant Pathology, Washington State University, Pullman, Washington 99164–6430, USA; 10 Department of Agriculture, Tottori University, Tottori, Tottori, 680–8553, Japan

**Keywords:** nonpathogenic *F. oxysporum*, phylogenetic analysis, *Solanum*, tomato, tomato wilt fungus

## Abstract

*Fusarium oxysporum* is an ascomycetous fungus that is well-known as a soilborne plant pathogen. In addition, a large population of nonpathogenic *F. oxysporum* (NPF) inhabits various environmental niches, including the phytosphere. To obtain an insight into the origin of plant pathogenic *F. oxysporum*, we focused on the tomato (*Solanum lycopersicum*) and its pathogenic *F. oxysporum* f. sp. *lycopersici* (*FOL*). We collected *F. oxysporum* from wild and transition *Solanum* spp. and modern cultivars of tomato in Chile, Ecuador, Peru, Mexico, Afghanistan, Italy, and Japan, evaluated the fungal isolates for pathogenicity, VCG, mating type, and distribution of *SIX* genes related to the pathogenicity of *FOL*, and constructed phylogenies based on ribosomal DNA intergenic spacer sequences. All *F. oxysporum* isolates sampled were genetically more diverse than *FOL*. They were not pathogenic to the tomato and did not carry *SIX* genes. Certain NPF isolates including those from wild *Solanum* spp. in Peru were grouped in *FOL* clades, whereas most of the NPF isolates were not. Our results suggested that the population of NPF isolates in *FOL* clades gave rise to *FOL* by gaining pathogenicity.

*Fusarium oxysporum* Schlecht. emend. Snyd. et Hans. is an ascomycetous fungus that inhabits various environments including the phytosphere, which includes both plant tissues and the rhizosphere. Most isolates from asymptomatic plants do not cause disease on any plants, and are referred to as nonpathogenic *F. oxysporum* (12).

On the other hand, plant pathogenic forms, formae speciales (f. spp.), are recognized in the species, and each form is defined by its strict host specificity (4, 5). *F. oxysporum* f. sp. *lycopersici* Snyd. et Hans. (*FOL*) is a pathogenic form that causes soilborne vascular wilt disease in the tomato (*Solanum lycopersicum* L.). Moreover, each of the three *FOL* pathogenic races (1, 2, 5) has been defined based on the possession of different combinations of SIX (secreted in xylem) protein genes, *SIX4*, *SIX3*, and *SIX1* (16, 17, 41), and determined by their specificities to particular tomato cultivars (2, 13, 53). These *SIX* genes are recognized to be pathogenic determinants and can be useful tools for race determination (18, 30).

“When, where, and how did plant pathogenic *F. oxysporum* emerge?” This is a very fundamental, but difficult question to address. Several phylogenetic studies have examined other plant pathogenic fungi using isolates from the places of origin and domestication of plants, for example, rice blast fungus *Pyricularia oryzae* Cavara [synonym, *Magnaporthe oryzae* (Hebert) Barr], late blight pathogen *Phytophthora infestans* (Mont.) de Bary, wheat fungal leaf blotch pathogen *Mycosphaerella graminicola* (Fückel) Schrot, and corn smut fungus *Ustilago maydis* (DC.) Corda (8, 14, 33, 48). To date, phylogenetic studies have also been extensively performed on *F. oxysporum* isolates (9, 12, 21–23, 29, 32, 34). For example, *FOL* is considered to be polyphyletic because it is composed of isolates involved in three clades (19, 23), and the pathogen of Fusarium wilt of melon (f. sp. *melonis*) has also been shown to be polyphyletic (12), whereas the cabbage yellows fungus (f. sp. *conglutinans*) is composed of one cluster and appears to be monophyletic (22). Studies on pathogenic isolates are generally limited, and very little is known about the relationship between pathogenic and nonpathogenic isolates. Therefore, we focused on the co-evolution of the tomato wilt pathogen and tomato.

The tomato (*S. lycopersicum*) is thought to have originated in South America, which is now occupied by Peru, Chile, Ecuador, and Bolivia. This region continues to sustain wild species of *Solanum* L. section *Lycopersicon* (Miller) Wettstein, such as *S. cheesmaniae* (Riley) Fosberg (syn. *Lycopersicon cheesmaniae* Riley), *S. chilense* (Dunal) Reiche (syn. *L. chilense* Dunal), *S. chmielewskii* Rick *et al.* (syn. *L. chmielewskii* Rick *et al.*), *S. galapagense* Darwin et Peralta (syn. *L. cheesmaniae* Riley), *S. habrochaites* Knapp et Spooner (syn. *L. hirsutum* Dunal), *S. neorickii* Spooner *et al.* (syn. *L. parviflorum* Rick *et al.*), *S. pennellii* Correll (syn. *L. pennellii* [Correll] D’Arcy), *S. peruvianum* L. (syn. *L. peruvianum* [L.] Miller), and *S. pimpinellifolium* L. (syn. *L. pimpinellifolium* [L.] Miller) (39).

A wild *Solanum* sp., possibly *S. pimpinellifolium*, spread prehistorically from South America to Central America (Mexico) in which the tomato was domesticated (20). *S. lycopersicum* var. *cerasiforme*, an apparent intermediate between wild and cultivated tomatoes (42), is currently found as a natively grown (“silvestre” in Spanish) tomato in some rural areas of Mexico. Traditional tomato cultivars, so-called “jitomate criollo” in Spanish, have been handed down by generations of peasants in mountain villages, and are considered the archetype of modern tomatoes due to their diverse morphologies (20). *S. lycopersicum* var. *cerasiforme* and jitomate criollo were designated transition tomatoes in this study. Tomatoes were transported to European countries, such as Italy and Spain, in which modern tomato breeding started, during the Spanish conquest in the 16th century (20, 39, 50).

In the present study, we 1) collected *F. oxysporum* isolates from tissues and the rhizosphere of asymptomatic *Solanum* biotypes: wild tomatoes in Chile, Ecuador, and Peru; transition tomatoes in Mexico; and modern tomatoes worldwide, 2) evaluated the pathogenicity of each isolate by an inoculation test using tomato tester cultivars, 3) evaluated the susceptibility of each *Solanum* biotype to *FOL* by the inoculation test, 4) determined the mating type and VCG of each isolate, 5) performed phylogenetic analyses based on sequences of the ribosomal DNA intergenic spacer (rDNA-IGS) region of the *F. oxysporum* isolates, together with *FOL* and other f. spp. collected worldwide, and 6) detected *SIX* genes in the *F. oxysporum* isolates collected. Based on the results obtained, we attempted to determine when, where, and how the plant pathogenic forms of *F. oxysporum* emerged.

## Materials and Methods

### Plant tissues and rhizosphere soil samples

We sampled the leaves, flowers, stems, fruits, roots, and rhizosphere soils of asymptomatic *Solanum* (sect. *Lycopersicon*) spp. in Chile, Peru, Ecuador, Mexico, Italy, Afghanistan, and Japan between 2002 and 2011 (Table 1). Here, rhizosphere soil refers to soil sampled from an area *ca.* 5 cm from the plant base and the surface at a depth of *ca.* 5 cm.

### Isolation of *F. oxysporum* from plant tissues and rhizosphere soil

Fungal isolations were prepared within 10 d of collecting the *Solanum* tissues. Small pieces (*ca.* ≤ 9 mm^2^) from individual tissue samples were cut and placed on *Fusarium*-selective media (25, 35) and potato sucrose agar (PSA) medium in a Petri dish, and incubated at 28°C in the dark.

Fungal isolations from the rhizosphere were prepared by the soil-plate method (54) using *Fusarium*-selective media. Briefly, approximately 0.5 g of a soil sample was dispersed in 15 mL molten medium in a Petri dish and then incubated at 28°C under dark.

Fungal colonies that emerged after the 2–4-d incubation were transferred onto fresh medium and purified by repeated single hyphal tip isolation. Each established isolate was maintained on a PSA plate at 28°C, and isolates identified as *F. oxysporum* based on morphological characteristics (28) were subjected to further studies. All the isolates were stored in 25% glycerol solution at −150°C.

### Inoculation test

The pathogenicity of each *F. oxysporum* isolate was evaluated using tomato tester cultivars. To prepare the inoculum, each isolate was cultured for 5 d on 3 mL potato dextrose broth (PDB; Becton and Dickinson, MA, USA) in a 15-mL screw cap test-tube at 25°C on a reciprocal shaker (Taitec, Saitama, Japan) at 200 strokes min^−1^. Budding cells were collected by centrifugation (3000×*g*, 15 min) and adjusted to ≥1.0×10^7^ cells mL^−1^. *FOL* MAFF 305121 (race 1), JCM 12575 (race 2), and Chz1-A (race 3) were used as positive controls in this assay.

Three tomato standard tester cvs. Ponderosa (*i i2 i3*, susceptible to all *FOL* races; Takayama Seed, Kyoto, Japan), Momotaro (*I i2 i3*, resistant to race 1 and susceptible to races 2 and 3; Takii seeds, Kyoto, Japan), and Walter (*I I2 i3*, resistant to races 1 and 2 and susceptible to race 3; gift from the National Institute of Vegetable and Tea Science, Mie, Japan) were used (3). Two seeds were sown for each test in sterilized soil (andosol) in a plastic pot (7 cm in diameter) and were grown in a greenhouse at 28°C.

Prior to the inoculation, the roots of 2–3-week-old plants were injured by repeatedly inserting a plastic peg into the soil. The inoculum (2 mL pot^−1^) was poured on the soil surface and allowed to soak into the rhizosphere. After a month, the external symptoms of each plant were evaluated as follows: 0, no wilt or yellowing; 1, lower leaves yellowing; 2, lower and upper leaves yellowing; 3, lower leaves yellowing and wilting, and upper leaves yellowing; 4, all leaves wilting and yellowing, or dead.

### Susceptibility of collected wild and transitional *Solanum* spp. to *FOL*

A part of the *Solanum* spp. germ collection was used to evaluate susceptibility to *FOL* MAFF 305121 (race 1), JCM 12575 (race 2) and Chz1-A (race 3); *S. chilense* Lc0036 (Chile/S18°27′16.3″/W69°46′22.1″/altitude, 2460 m), *S. peruvianum* Lp0043-1 (Chile/S18°24′42.8″/W70°12′43.8″/altitude, 211 m), *S. peruvianum* Lp0044 (Chile/S18°24′43.9″/W70°12′06.2″/altitude, 233 m), *S. peruvianum* Lp0046 (Chile/S18°25′03.6″/W70°06′13.3″/altitude, 410 m), *S. pimpinellifolium* Lpp0040 (Ecuador/S00°39′03.8″/W90° 24′12.9″/altitude, 432 m), *S. pimpinellifolium* Lpp0041w1 (Ecuador/S00°41′27.1″/W90°19′21.9″/altitude, 189 m), *S. pimpinellifolium* Lpp0043 (Ecuador/S00°41′23.0″/W90°19′10.3″/altitude, 208 m), *S. pimpinellifolium* Lpp0045 (Ecuador/S00°40′05.2″/W90°16′08.9″/altitude, 253 m), *S. lycopersicum* var. *cerasiforme* Lec0001 (Mexico/N20°24′21.4″/W89°45′25.2″/altitude, 40 m), *S. lycopersicum* (jitomate criollo) Lecr0001 (Mexico/N17°24′22.4″/W92°02′01.0″/altitude, 400 m). Each of these plants was prepared as described above, and the inoculation with *FOL* races 1–3 was performed after the third leaf appeared. After a month, the inner symptoms of each plant were evaluated as follows: 0, no vascular browning; 1, browning in 1–25% of vascular; 2, browning in 26–50% of vascular; 3, browning in 51–75% of vascular; 4, browning in 75–100% of vascular.

### Fungal DNA extraction

Genomic DNA (gDNA) was extracted from fungal mycelia following a protocol modified from the original method (45). Briefly, a small amount of mycelia on PSA medium (≤25 mm^2^) was placed in 500 μL lysis buffer (50 mM EDTA, 200 mM NaCl, 1% *n*-lauroylsarcosine sodium salt, 200 mM Tris-HCl pH 8.0) in a microtube, incubated for 10 min at room temperature, centrifuged at 20,000×*g* for 5 min at 4°C after the addition of 150 μL of 3 M potassium acetate. The supernatant was then transferred to a fresh microtube. gDNA in the supernatant was concentrated by ethanol precipitation and resuspended in TE buffer (10 mM Tris-HCl pH 8.0, 1 mM EDTA).

### Polymerase chain reaction (PCR)

A standard reaction mixture (20 μL) contained 20 ng gDNA, 2 μL 10×buffer (Takara Bio, Otsu, Japan), 1.6 μL of 2.5 mM (each) dNTPs (Takara Bio), 8 pM of each primer, and 0.5 U of Ex-Taq polymerase (Takara Bio) or 5 μL of GoTaq^®^ Master Mix (Promega, Madison, WI, USA). The primers used in this study are listed in Table 2.

To identify *F. oxysporum* and perform a phylogenetic analysis, a part of the rDNA-IGS region (*ca.* 600 bp) was amplified using the primer set FIGS11/FIGS12 (22). The mating type (MAT1-1 or MAT1-2) of each isolate was determined using primer sets Gfmat1a/Gfmat1b and GfHMG1/GfHMG2 (19). The presence of *SIX4*, *SIX3* and *SIX1* genes in each isolate was determined using the primer sets SIX4F/SIX4R, SIX3-F2/SIX3-R1, and P12-F2/P12-R1, respectively (Table 2).

### DNA sequencing

The IGS amplicons of rDNA-IGS from *F. oxysporum* were purified with EXOSAP-IT (USB, Cleveland, OH, USA) and sequenced with a 3130xl Genetic Analyzer (Applied Biosystems, Foster City, CA, USA) using the BigDye^®^ Terminator v1.1/v3.1 Cycle Sequencing kit (Applied Biosystems) and the primer set FIGS11/FIGS12 (22). Sequences were deposited in GenBank (http://www.ncbi.nlm.nih.gov/Database/), where they were assigned accession numbers (Table 1).

### Phylogenetic analyses

Nucleotide sequences were arranged with GENETYX-MAC ver.10.1/13 (Genetyx, Tokyo, Japan) and aligned with the sequences of other *Fusarium* isolates (Table 3) using CLUSTALX v.2.0 (26). All gaps in the alignment were ignored in subsequent analyses.

*F. oxysporum* phylogenies were estimated using three methods including maximum likelihood (ML) (10), maximum parsimony (MP) (11), and Bayesian inference (BI) (55). All of the following *F. oxysporum* phylogenies were rooted with *F. sacchari* strain FGSC 7610 (Table 3) as the outgroup.

ML phylogenies were estimated using RAxML implemented in raxmlGUI 1.0 (46). MrModeltest v2.3 (36) determined the appropriate substitution model as the HKY+G model from the model of the hierarchical likelihood ratio test (hLRT). Although the HKY+G model was not implemented in raxmlGUI, the HKY+G model was displaceable by the GTR model (A. Stamatakis, pers. comm.); therefore, the analysis was performed with the GTRGAMMA model and rapid bootstrap option (47) with 1,000 bootstrap replicates.

In the MP analysis using PAUP* 4.0b10 (49), searches of trees included 1,000 random additions, heuristic replicates with tree bisection, and reconnection (TBR) branch-swapping. One thousand bootstrap replicates were performed with the heuristic search option.

BI phylogenies were estimated using MrBayes 3.1.2 (43) based on the HKY+G model. In the BI analysis, the Markov Chain Monte Carlo (MCMC) iterations with four chains were started from a random tree topology and lasted 500,000,000 generations. When the average standard deviation of the split frequencies was below 0.01, the MCMC iterations were stopped automatically. Trees were saved at each 100-generation interval, and 12,500 trees were discarded as burn-in. Finally, the posterior probabilities of each branch were calculated.

### Vegetative compatibility group (VCG) typing

VCG reflects genetic variations among fungal isolates (40). Four VCGs (0030+0032, 0031, 0033 and 0035) have been reported previously in *FOL* (6), and these have correlated with phylogeny (23, 32). The following *FOL* tester isolates: OSU-451B (VCG 0031), MN-66 (VCG 0030+0032), and H-1-4 (VCG 0033) were used to determine the VCG of each isolate. The basis of the VCG test was as follows; by a selection on MMC (minimal agar medium with 1.5% chlorate), a mutation (at either *nit1* or NitM) causing nitrate nonutilization was introduced into each collected isolate to be tested and into each of the three tester strains. The mutation in each tester was assessed using hypoxanthine medium (0.2 g L^−1^ of hypoxanthine plus minimal agar medium without NaNO_3_; *nit1* +, NitM −) and nitrite medium (0.5 g L^−1^ of NaNO_2_ plus minimal agar medium without NaNO_3_; *nit1* +, NitM +). To assess VCGs, a part of the collected isolates was paired on MM (minimal medium) with *nit*-complementary testers; *nit-*complementary testers were paired with each other as positive controls. Vigorous growth on MM reflected heterokaryon formation, which indicating that the paired isolates belonged to the same VCG of the tester (7).

## Results

### Sampling of *Solanum* spp. and isolation of fungi from plant tissue and rhizosphere soil

Among the wild tomatoes, *S. chilense* was sampled in Chile and Peru, *S. habrochaites* was sampled in Peru, *S. pennellii* was sampled in Peru, *S. peruvianum* was sampled in Chile and Peru, and *S. pimpinellifolium* was sampled in Peru and Ecuador. Transition tomatoes were sampled in Mexico. The Mexican transition tomatoes were morphologically diverse; the colors of mature fruits were red, orange, or yellow. In addition, jitomate criollo fruits had irregular multiloculated shapes and were heterogeneous in size (Fig. S1i, j). Modern tomatoes cultivated in farmlands were sampled in Chile, Mexico, Italy, Afghanistan, and Japan. None of the plants exhibited wilt symptoms at the time of collection. The precise locations (latitude, longitude, and altitude) of each collection field and plant sample are presented in Table 1 and Fig. S1a–j.

Approximately 2,500 fungal isolates were obtained from the plant and rhizosphere soil samples. Based on the morphological characteristics and nucleotide sequences of IGS regions, 433 of these isolates were identified as *F. oxysporum*; 42 were from plant tissues and 391 were from rhizosphere soils. *F. oxysporum* was not isolated from the tissues of *S. chilense*. A multitude of other fungi were also recovered from plant tissues and rhizosphere soils, *e.g.* mitosporic ascomycetes such as *Fusarium* spp., *Trichoderma* spp., *Penicillium* spp., *Cladosporium* spp., *Alternaria* spp., and *Phoma* spp., and zygomycetes such as *Mucor* spp.

### *F. oxysporum* pathogenicity assay

**Fig. 1:**
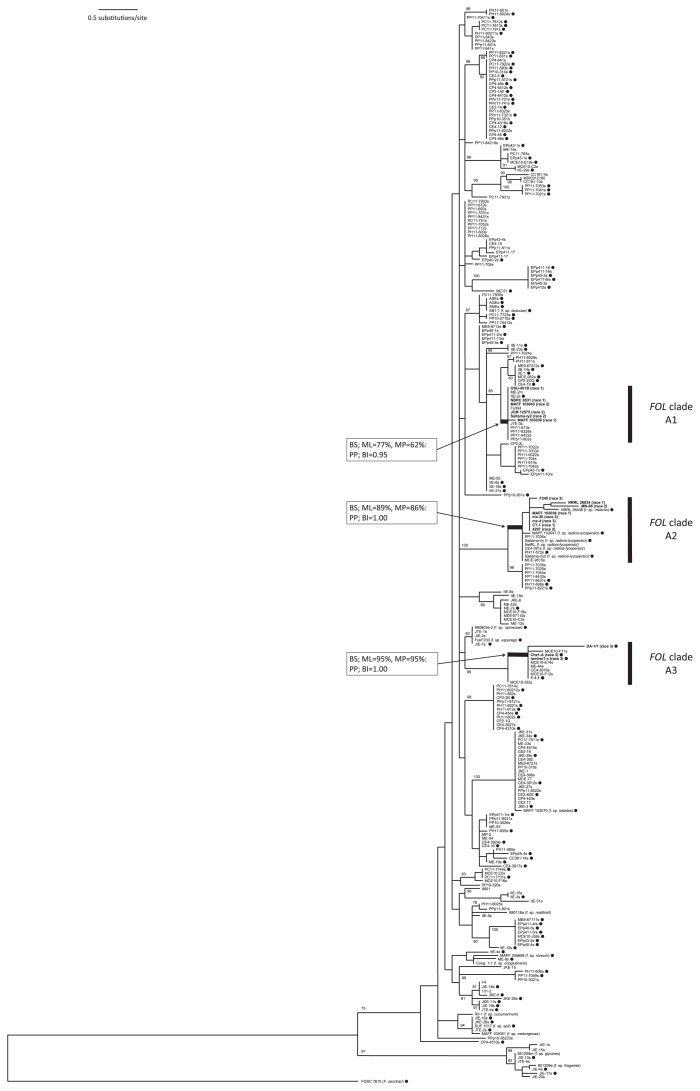
Maximum likelihood (ML) tree based on the intergenic spacer (IGS) region of *Fusarium oxysporum* isolates estimated using raxmlGUI 1.0 (46). *F. sacchari* strain FGSC 7610 was used as the outgroup. Bootstrap values (1,000 bootstrapped datasets) calculated in the ML analysis as greater than 60% are shown beside the branches. The *FOL* clusters A1, A2, and A3 (shown in black bars) are identical to those reported in a previous study (23), and bootstrap values in maximum likelihood (ML)/maximum parsimony (MP) analyses and posterior probability values in BI analysis are shown on the three clades only. *FOL* isolates and their clades are shown in bold characters with their race in parentheses. Filled circles show MAT1-2 isolates.

None of the 433 *F. oxysporum* isolates, except for CE-391s, caused wilt disease when inoculated on the three tomato tester cultivars. We designated the *F. oxysporum* isolates that did not cause wilt on the tomato as NPF in this study (Table 1). CE4-391s was isolated from the rhizosphere soil of a modern tomato cultivar in a Chilean tomato farmland, and caused crown and root rot symptoms (27) on all three tester cultivars (Table 3). The IGS sequence of CE4-391s was identical to that of *F. oxysporum* Schlecht. f. sp. *radicis-lycopersici* Jarvis et Shoem. (*FORL*) strain Saitamarly (Fig. 1, Table 3), a known crown and root rot pathogen of the tomato. These results, along with the finding that CE4-391s lacked *SIX* genes that are unique to *FOL* (52), led us to conclude that CE4-391s was neither NPF nor *FOL*, but rather *FORL*.

### Phylogenetic analyses

Among the 432 NPFs identified, several isolates from the same sample and carrying identical rDNA-IGS sequences, were considered clonal, and one of them was used as their representative for phylogenetic studies. Therefore, phylogenetic trees were estimated using 233 NPFs (Table 1), together with 18 *FOL* isolates, 18 isolates of other formae speciales, and 5 NPFs isolated in previous studies (Table 3).

Maximum likelihood (ML), maximum parsimony (MP), and Bayesian inference (BI) methods were used to construct phylogenetic trees, and the ML tree was shown in Fig. 1. The topology of the ML tree was nearly identical to those of the MP and BI trees (data not shown). Each branch was statistically estimated by a bootstrap (BS) test in ML and MP analyses, and posterior probability (PP) in BI analysis. The parameter of the ML tree (−ln *L* = 3419.861497) was as follows; base frequencies = (A = 0.159040, C = 0.175477, G = 0.363070, T = 0.302413). MP analysis yielded 1,000 equally parsimonious trees (tree length = 413 steps; consistency index = 0.741; retention index = 0.929; rescaled consistency index = 0.688; homoplasy index = 0.259).

In the ML tree, *FOL* isolates were found in three clades (A1, A2, and A3; indicated in black bars in Fig. 1). This was also the case for MP and BI trees (data not shown). These results were consistent with the findings of previous studies (23, 38), in which *FOL* was shown to be polyphyletic. In these *FOL* clades, not only *FOL* isolates, but also 16 NPF isolates (8 for the A1 clade, 3 for the A2 clade, and 5 for the A3 clade) were grouped. Within each clade, the IGS sequences of NPF were 99.8 to 100% identical to those of *FOL*.

The A2 clade was supported (BS; ML = 89%, MP = 86%: PP; BI = 1.00), in which three NPF isolates, PP11-7035s (from the rhizosphere of *S. peruvianum*, Peru), PH11-572s (from the rhizosphere of *S. habrochaites*, Peru), and MCE-9515s (from the rhizosphere of *S. lycopersicum* var. *cerasiforme*, Mexico), were grouped together with *FOL* (F240, NRRL 26034, MN-66, MAFF 103036, mx-20, mx-4, CT-1, and 4287) and also *FORL* isolates. The A3 clade was supported well (BS; ML = 95%, MP = 95%: PP; BI = 1.00), in which five NPF isolates, ME-44s (from the rhizosphere of jitomate criollo, Mexico), CE4-3916s (from the rhizosphere of *S. lycopersicum*, Chile), MCE10-E14s (from the rhizosphere of *S. lycopersicum* var. *cerasiforme*, Mexico), MCE10-F11s (from the rhizosphere of *S. lycopersicum* var. *cerasiforme*, Mexico), and MCE10-F12s (from the rhizosphere of *S. lycopersicum* var. *cerasiforme*, Mexico), were grouped with *FOL* isolates (DA-1/7, Chz1-A, tomato1-c and F-1-1). The A1 clade included eight NPF isolates, PH11-613s (from the rhizosphere of *S. habrochaites*, Peru), PP11-8328s (from the rhizosphere of *S. peruvianum*, Peru), PP11-8422s (from the rhizosphere of *S. peruvianum*, Peru), PPp11-802s (from the rhizosphere of *S. pimpinellifolium*, Peru), ME-2m (from *S. lycopersicum* jitomate criollo fruit, Mexico), Fo304 (from the rhizosphere of *S. lycopersicum*, Japan), JTE-3s (from the rhizosphere of *S. lycopersicum*, Japan), and ItE-2s (from the rhizosphere of *S. lycopersicum*, Italy), together with *FOL* isolates (OSU-451B, NBRC 6531, MAFF 103043, JCM 12575, Saitama-ly2, and MAFF 103038). This A1 clade was less supported (BS; ML = 77, MP = 62: PP; BI = 0.95) than the A2 and A3 clades. However, the A1 clade was reproducible in ML, MP, and BI phylogenies, which indicated that the isolates in the A1 clade as well as those in the A2 and A3 clades were monophyletic.

These 16 NPF isolates in the *FOL* clades were obtained from Peruvian wild species of tomatoes, Mexican transitional tomatoes and modern tomato cultivars worldwide, while none of the NPF isolates were obtained from wild species in Chile and Ecuador.

### Mating type and VCG determination

Among the 432 NPFs, 184 and 243 isolates were MAT1-1 and MAT1-2, respectively (5 isolates were not tested). Homothallic (*MAT1-1* + *MAT1-2*) isolates were not detected.

We tested vegetative compatibility between *FOL* and the subset of 16 NPF isolates from our fungal collection that fell into the three *FOL* clades (Fig. 1). Although each of the NPFs was paired with the VCG 0031, 0030+0032, and 0033 tester strains, none were compatible.

### Tests for *SIX* genes

PCR analyses indicated that the 16 NPF isolates that grouped into the *FOL* clades did not carry *SIX1*, *SIX3*, or *SIX4*. These genes were readily amplified from the authentic *FOL* strains.

### *Solanum* spp. susceptibility assay

The Mexican transition tomatoes, *S. lycopersicum* var. *cerasiforme* and *S. lycopersicum* (jitomate criollo), showed an almost equivalent degree of susceptibility to that of cv. Ponderosa (a modern tomato cultivar carrying no resistance) to *FOL* races 1–3. Among the wild species of tomatoes, *S. chilense* and *S. peruvianum* showed resistance to *FOL* races 1–3 (Table 4). On the other hand, the resistance of all *S. pimpinellifolium* collections from Ecuador was less than that of the above two wild species (Table 4), although they presented no external symptoms.

## Discussion

It has generally been assumed that a plant pathogen emerged from a nonpathogenic strain during the domestication and breeding of its host plants. Several previous studies (8, 14, 33, 48) suggested a relationship between the origin of pathogens and domestication of host plants. However, such studies have not yet been performed on *Fusarium oxysporum*.

In the present study, we isolated *F. oxysporum* from the tissues and rhizosphere soils of asymptomatic *Solanum* spp. sect. *Lycopersicon* and found that all the *F. oxysporum* isolates recovered were nonpathogenic *F. oxysporum* (NPF), except for one isolate (CE4-391s) from a modern tomato field in Chile, which was considered to be *FORL*. This result was consistent with the findings of previous studies (12), which showed that NPFs were frequently isolated from plants and, therefore, are part of the normal field population. In our phylogeny, *FOL* isolates were distributed in any of the three clades (A1, A2, and A3; Fig. 1), suggesting that *FOL* has at least three origins (polyphyletic), which is consistent with the findings of previous studies (23, 37, 38). We also found that 16 NPFs were grouped in the three *FOL* clades (3 for the A2 clade, 8 for the A1 clade and 5 for the A3 clade), and that they are more closely related to *FOL* (99.8 to 100% nucleotide identity of rDNA-IGS) than to other NPFs and isolates of other forms (82.0 to 99.5% nucleotide identity). These 16 NPFs were isolated from Peruvian wild species, transition tomatoes, or modern cultivars. This result suggests that these NPFs share common ancestors with *FOL* and that the possible origin of *FOL* existed with the wild *Solanum* spp. in the Andes, possibly in Peru.

How did *FOL* acquire pathogenicity to the tomato? Kistler proposed a horizontal gene transfer (HGT) to explain the evolution of pathogenicity in *F. oxysporum* (24). HGT or horizontal chromosomal transfer (HCT) has been reported in other plant pathogenic fungi, such as *Nectria haematococca* (15), *Cochliobolus heterostrophus* (44), and *Alternaria alternata* (1). A small (*ca.* 2.0 Mb) chromosome, designated chromosome 14 (Ch14), was recently detected on *FOL* (31), and was found to carry effector genes, such as *SIX1*, *SIX3*, *SIX4* and other genes presumably related to pathogenicity (19, 51). *FOL* isolates belonging to each distinct *FOL* clade in the phylogeny shared genes (Fig. 1). These results suggest that *FOL* had a polyphyletic origin, and that the original NPF may have acquired the small chromosome involved in pathogenicity and/or host specificity of *FOL* by HCT.

The detailed mechanisms underlying HCT and HGT in fungi are unclear (51). However, Ma and co-workers demonstrated detected HCT in *F. oxysporum in vitro* (31). They co-incubated the pathogenic *FOL* strain Fol007 (possessing Ch14) with the NPF strain Fo-47 (lacking Ch14), and recovered a Fo-47 bearing Ch14 that presented pathogenicity to the tomato. Ch14 could only be transferred to strain Fo-47, but not to *F. oxysporum* f. sp. *melonis* or *F. oxysporum* f. sp. *cubense*, by the same manner. This experiment suggested that HGT or HCT may not occur randomly among strains, but rather depends on particular strains or environmental conditions. To test this foregoing hypothesis, it will be necessary to demonstrate that the 16 NPF isolates in the *FOL* clades (Fig. 1) have a greater capacity to acquire the small chromosome carrying effector genes than other more distantly related isolates.

The results of this study suggest that the nonpathogenic ancestors of *FOL* were in Peru, and a part of their progenitors gained effector genes or the small chromosome later, which resulted in the emergence of *FOL*. The origin(s) of the effector genes carried by the small chromosome are of interest. Mexican transitional tomatoes and modern cultivars are less resistant to *FOL* than wild species (Table 4); therefore, clear damage by *FOL* may have appeared during/after tomato domestication in Mexico.

Our study represents an initial step in an investigation to discover the origin of *FOL*. We are now interested in examining the origin of the pathogenicity determinants/Ch14 in *FOL* (31). Studies on the distribution of resistance genes (*I*–*I3*) among tomatoes, *Solanum* section *Lycopersicon*, are also warranted. Our goal is to advance our understanding on the molecular mechanisms underlying host-parasite co-evolution.

## Supplementary Information



## Figures and Tables

**Table 1:** *Fusarium oxysporum* isolated from the tissue and rhizosphere of *Solanum* spp. (sec. *Lycopersicon*)

Name	Source of fungal isolates	Collected site	Year	Country/latitude/longitude/altitude^a^	Mating type	GenBank Accession No.
***F. oxysporum*** **from wild species**						
CC161-4s	*S. chilense*	soil	2004	Chile/S18°28′01.8″/W69°49′27.5″/1939 m	1-1	AB373843
CC161-12s	*S. chilense*	soil	2004	Chile/S18°28′3.0″/W69°49′45.9″/1939 m	1-1	AB373844
CC361-14s	*S. chilense*	soil	2004	Chile/S18°27′16.3″/W69°46′22.1″/2460 m	1-2	AB373845
PC11-751s	*S. chilense*	soil	2011	Peru/S16°59′04.0″/W71°46′14.2″/924 m	1-1	AB697899
PC11-7611s	*S. chilense*	soil	2011	Peru/S17°09′47.4″/W70°52′21.4″/1653 m	1-2	AB697900
PC11-7612s	*S. chilense*	soil	2011	Peru/S17°09′47.4″/W70°52′21.4″/1653 m	1-2	AB697901
PC11-7613s	*S. chilense*	soil	2011	Peru/S17°09′47.4″/W70°52′21.4″/1653 m	1-2	AB697902
PC11-7614s	*S. chilense*	soil	2011	Peru/S17°09′47.4″/W70°52′21.4″/1653 m	1-1	AB697903
PC11-7723s	*S. chilense*	soil	2011	Peru/S17°08′48.1″/W70°51′24.2″/1795 m	1-2	AB697904
PC11-7731s	*S. chilense*	soil	2011	Peru/S17°08′48.1″/W70°51′24.2″/1795 m	1-2	AB697905
PC11-7744s	*S. chilense*	soil	2011	Peru/S17°08′48.1″/W70°51′24.2″/1795 m	1-2	AB697906
PC11-783s	*S. chilense*	soil	2011	Peru/S17°07′32.6″/W70°50′25.9″/1912 m	1-1	AB697907
PC11-791s	*S. chilense*	soil	2011	Peru/S17°06′46.8″/W70°50′32.3″/2014 m	1-2	AB697908
PC11-7922s	*S. chilense*	soil	2011	Peru/S17°06′46.8″/W70°50′32.3″/2014 m	1-2	AB697909
PC11-7931s	*S. chilense*	soil	2011	Peru/S17°06′46.8″/W70°50′32.3″/2014 m	1-1	AB697910
PC11-7933s	*S. chilense*	soil	2011	Peru/S17°06′46.8″/W70°50′32.3″/2014 m	1-1	AB697911
PC11-7935s	*S. chilense*	soil	2011	Peru/S17°06′46.8″/W70°50′32.3″/2014 m	1-1	AB697912
CP2-2L	*S. peruvianum*	stem	2002	Chile/S18°25′02.1″/W70°06′02.9″/436 m	1-1	AB373833
CP2-1G	*S. peruvianum*	stem	2002	Chile/S18°24′32.7″/W70°12′20.0″/215 m	1-1	AB373835
CP2-2B	*S. peruvianum*	stem	2002	Chile/S18°25′02.1″/W70°06′02.9″/436 m	1-2	AB373836
CP2-2OQ	*S. peruvianum*	stem	2002	Chile/S18°25′02.1″/W70°06′02.9″/436 m	1-2	AB373837
CP4-46	*S. peruvianum*	stem	2004	Chile/S18°25′03.6″/W70°06′13.3″/410 m	1-2	AB373846
CP2-1AF	*S. peruvianum*	fruit	2002	Chile/S18°24′32.7″/W70°12′20.0″/215 m	1-2	AB373834
CP4-45	*S. peruvianum*	fruit	2004	Chile/S18°25′35.4″/W70°06′19.6″/408 m	1-2	AB373847
CP4-4310s	*S. peruvianum*	soil	2004	Chile/S18°24′42.8″/W70°12′43.8″/211 m	1-2	AB373852
CP4-4318s	*S. peruvianum*	soil	2004	Chile/S18°24′42.8″/W70°12′43.8″/211 m	1-2	AB373848
CP4-441s	*S. peruvianum*	soil	2004	Chile/S18°24′43.9″/W70°12′06.2″/233 m	1-1	AB373855
CP4-4412s	*S. peruvianum*	soil	2004	Chile/S18°24′43.9″/W70°12′06.2″/233 m	1-2	AB373849
CP4-4510s	*S. peruvianum*	soil	2004	Chile/S18°25′35.4″/W70°06′19.6″/408 m	1-2	AB373857
CP4-4512s	*S. peruvianum*	soil	2004	Chile/S18°25′35.4″/W70°06′19.6″/408 m	1-2	AB373850
CP4-4515s	*S. peruvianum*	soil	2004	Chile/S18°25′35.4″/W70°06′19.6″/408 m	1-1	AB373856
CP4-453s	*S. peruvianum*	soil	2004	Chile/S18°25′35.4″/W70°06′19.6″/408 m	1-1	AB373854
CP4-454s	*S. peruvianum*	soil	2004	Chile/S18°25′35.4″/W70°06′19.6″/408 m	1-2	AB373853
CP4-46s	*S. peruvianum*	soil	2004	Chile/S18°25′03.6″/W70°06′13.3″/410 m	1-2	AB373851
PP10-2710s	*S. peruvianum*	soil	2010	Peru/S11°46′18.7″/W76°18′15.0″/3038 m	1-2	AB627132
PP10-3021s	*S. peruvianum*	soil	2010	Peru/S11°48′24.6″/W76°20′27.5″/2595 m	1-1	AB627133
PP10-3026s	*S. peruvianum*	soil	2010	Peru/S11°48′24.6″/W76°20′27.5″/2595 m	1-1	AB627134
PP10-314s	*S. peruvianum*	soil	2010	Peru/S11°49′07.7″/W76°21′25.1″/2499 m	1-2	AB627135
PP10-316s	*S. peruvianum*	soil	2010	Peru/S11°49′07.7″/W76°21′25.1″/2499 m	1-1	AB627136
PP10-326s	*S. peruvianum*	soil	2010	Peru/S11°51′23.9″/W76°25′10.0″/2225 m	1-1	AB627137
PP11-672s	*S. peruvianum*	soil	2011	Peru/S16°24′57.2″/W71°28′02.5″/2682 m	1-1	AB697913
PP11-692s	*S. peruvianum*	soil	2011	Peru/S16°24′33.2″/W71°27′03.1″/2830 m	1-1	AB697914
PP11-704s	*S. peruvianum*	soil	2011	Peru/S16°17′41.0″/W71°38′53.2″/2632 m	1-1	AB697915
PP11-705s	*S. peruvianum*	soil	2011	Peru/S16°17′41.0″/W71°38′53.2″/2632 m	1-1	AB697916
PP11-7021s	*S. peruvianum*	soil	2011	Peru/S16°17′41.0″/W71°38′53.2″/2632 m	1-2	AB697917
PP11-7022s	*S. peruvianum*	soil	2011	Peru/S16°17′41.0″/W71°38′53.2″/2632 m	1-1	AB697918
PP11-7024s	*S. peruvianum*	soil	2011	Peru/S16°17′41.0″/W71°38′53.2″/2632 m	1-1	AB697919
PP11-7025s	*S. peruvianum*	soil	2011	Peru/S16°17′41.0″/W71°38′53.2″/2632 m	1-1	AB697920
PP11-7031s	*S. peruvianum*	soil	2011	Peru/S16°17′41.0″/W71°38′53.2″/2632 m	1-1	AB697921
PP11-7033s	*S. peruvianum*	soil	2011	Peru/S16°17′41.0″/W71°38′53.2″/2632 m	1-1	AB697922
PP11-7035s	*S. peruvianum*	soil	2011	Peru/S16°17′41.0″/W71°38′53.2″/2632 m	1-1	AB697923
PP11-7041s	*S. peruvianum*	soil	2011	Peru/S16°17′41.0″/W71°38′53.2″/2632 m	1-2	AB697924
PP11-7042s	*S. peruvianum*	soil	2011	Peru/S16°17′41.0″/W71°38′53.2″/2632 m	1-1	AB697925
PP11-7044s	*S. peruvianum*	soil	2011	Peru/S16°17′41.0″/W71°38′53.2″/2632 m	1-1	AB697926
PP11-7049s	*S. peruvianum*	soil	2011	Peru/S16°17′41.0″/W71°38′53.2″/2632 m	1-2	AB697927
PP11-70411s	*S. peruvianum*	soil	2011	Peru/S16°17′41.0″/W71°38′53.2″/2632 m	1-2	AB697928
PP11-70412s	*S. peruvianum*	soil	2011	Peru/S16°17′41.0″/W71°38′53.2″/2632 m	1-1	AB697929
PP11-7052s	*S. peruvianum*	soil	2011	Peru/S16°17′41.0″/W71°38′53.2″/2632 m	1-1	AB697930
PP11-7053s	*S. peruvianum*	soil	2011	Peru/S16°17′41.0″/W71°38′53.2″/2632 m	1-2	AB697931
PP11-712s	*S. peruvianum*	soil	2011	Peru/S16°17′44.6″/W71°38′29.0″/2642 m	1-1	AB697932
PP11-831s	*S. peruvianum*	soil	2011	Peru/S17°00′161″/W72°03′891″/177 m	1-2	AB697933
PP11-8321s	*S. peruvianum*	soil	2011	Peru/S17°00′161″/W72°03′891″/177 m	1-2	AB697934
PP11-8323s	*S. peruvianum*	soil	2011	Peru/S17°00′161″/W72°03′891″/177 m	1-1	AB697935
PP11-8328s	*S. peruvianum*	soil	2011	Peru/S17°00′161″/W72°03′891″/177 m	1-1	AB697936
PP11-841s	*S. peruvianum*	soil	2011	Peru/S16°58′142″/W72°04′003″/378 m	1-1	AB697937
PP11-843s	*S. peruvianum*	soil	2011	Peru/S16°58′142″/W72°04′003″/378 m	1-1	AB697938
PP11-8421s	*S. peruvianum*	soil	2011	Peru/S16°58′142″/W72°04′003″/378 m	1-2	AB697939
PP11-8422s	*S. peruvianum*	soil	2011	Peru/S16°58′142″/W72°04′003″/378 m	1-1	AB697940
PP11-8423s	*S. peruvianum*	soil	2011	Peru/S16°58′142″/W72°04′003″/378 m	1-1	AB697941
PP11-84219s	*S. peruvianum*	soil	2011	Peru/S16°58′142″/W72°04′003″/378 m	1-1	AB697942
PP11-8431s	*S. peruvianum*	soil	2011	Peru/S16°58′142″/W72°04′003″/378 m	1-1	AB697943
PP11-8433s	*S. peruvianum*	soil	2011	Peru/S16°58′142″/W72°04′003″/378 m	1-1	AB697944
MP-2	*S. peruvianum*^b^	leaf	2005	Mexico/N18°38′21.5″/W100°49′23.3″/217 m	nt^c^	AB373871
PH11-572s	*S. habrochaites*	soil	2011	Peru/S11°19′15.7″/W76°52′19.0″/1269 m	1-2	AB697945
PH11-582s	*S. habrochaites*	soil	2011	Peru/S11°19′12.4″/W76°52′17.8″/1265 m	1-1	AB697946
PH11-583s	*S. habrochaites*	soil	2011	Peru/S11°19′12.4″/W76°52′17.8″/1265 m	1-2	AB697947
PH11-585s	*S. habrochaites*	soil	2011	Peru/S11°19′12.4″/W76°52′17.8″/1265 m	1-1	AB697948
PH11-601s	*S. habrochaites*	soil	2011	Peru/S11°21′26.1″/W76°48′49.0″/1953 m	1-1	AB697949
PH11-602s	*S. habrochaites*	soil	2011	Peru/S11°21′26.1″/W76°48′49.0″/1953 m	1-2	AB697950
PH11-603s	*S. habrochaites*	soil	2011	Peru/S11°21′26.1″/W76°48′49.0″/1953 m	1-1	AB697951
PH11-605s	*S. habrochaites*	soil	2011	Peru/S11°21′26.1″/W76°48′49.0″/1953 m	1-2	AB697952
PH11-606s	*S. habrochaites*	soil	2011	Peru/S11°21′26.1″/W76°48′49.0″/1953 m	1-2	AB697953
PH11-608s	*S. habrochaites*	soil	2011	Peru/S11°21′26.1″/W76°48′49.0″/1953 m	1-2	AB697954
PH11-6021s	*S. habrochaites*	soil	2011	Peru/S11°21′26.1″/W76°48′49.0″/1953 m	1-2	AB697955
PH11-6022s	*S. habrochaites*	soil	2011	Peru/S11°21′26.1″/W76°48′49.0″/1953 m	1-1	AB697956
PH11-6024s	*S. habrochaites*	soil	2011	Peru/S11°21′26.1″/W76°48′49.0″/1953 m	1-2	AB697957
PH11-6025s	*S. habrochaites*	soil	2011	Peru/S11°21′26.1″/W76°48′49.0″/1953 m	1-1	AB697958
PH11-6026s	*S. habrochaites*	soil	2011	Peru/S11°21′26.1″/W76°48′49.0″/1953 m	1-1	AB697959
PH11-6029s	*S. habrochaites*	soil	2011	Peru/S11°21′26.1″/W76°48′49.0″/1953 m	1-1	AB697960
PH11-60210s	*S. habrochaites*	soil	2011	Peru/S11°21′26.1″/W76°48′49.0″/1953 m	1-1	AB697961
PH11-60211s	*S. habrochaites*	soil	2011	Peru/S11°21′26.1″/W76°48′49.0″/1953 m	1-2	AB697962
PH11-60212s	*S. habrochaites*	soil	2011	Peru/S11°21′26.1″/W76°48′49.0″/1953 m	1-2	AB697963
PH11-611s	*S. habrochaites*	soil	2011	Peru/S11°21′25.3″/W76°48′50.6″/1963 m	1-1	AB697964
PH11-612s	*S. habrochaites*	soil	2011	Peru/S11°21′25.3″/W76°48′50.6″/1963 m	1-2	AB697965
PH11-613s	*S. habrochaites*	soil	2011	Peru/S11°21′25.3″/W76°48′50.6″/1963 m	1-1	AB697966
PH11-614s	*S. habrochaites*	soil	2011	Peru/S11°21′25.3″/W76°48′50.6″/1963 m	1-1	AB697967
PPn11-721s	*S. pennellii*	soil	2011	Peru/S16°01′19.8″/W72°29′17.8″/703 m	1-2	AB697968
PPn11-7321s	*S. pennellii*	soil	2011	Peru/S16°01′11.5″/W72°29′14.2″/734 m	1-2	AB697969
PPn11-741s	*S. pennellii*	soil	2011	Peru/S16°01′15.8″/W72°29′15.8″/709 m	1-2	AB697970
EPp411-17	*S. pimpinellifolium*	stem	2008	Ecuador/S00°41′27.1″/W90°19′21.9″/189 m	1-1	AB515354
EPp411-16	*S. pimpinellifolium*	root	2008	Ecuador/S00°41′27.1″/W90°19′21.9″/189 m	1-2	AB515353
EPp40-1s	*S. pimpinellifolium*	soil	2008	Ecuador/S00°39′03.8″/W90°24′12.9″/432 m	1-1	AB515355
EPp40-2s	*S. pimpinellifolium*	soil	2008	Ecuador/S00°39′03.8″/W90°24′12.9″/432 m	1-2	AB515356
EPp40-3s	*S. pimpinellifolium*	soil	2008	Ecuador/S00°39′03.8″/W90°24′12.9″/432 m	1-1	AB515357
EPp40-4s	*S. pimpinellifolium*	soil	2008	Ecuador/S00°39′03.8″/W90°24′12.9″/432 m	1-2	AB515358
EPp40-5s	*S. pimpinellifolium*	soil	2008	Ecuador/S00°39′03.8″/W90°24′12.9″/432 m	1-1	AB515359
EPp411-1rs	*S. pimpinellifolium*	soil	2008	Ecuador/S00°41′27.1″/W90°19′21.9″/189 m	1-2	AB515360
EPp411-2rs	*S. pimpinellifolium*	soil	2008	Ecuador/S00°41′27.1″/W90°19′21.9″/189 m	1-2	AB515361
EPp411-4rs	*S. pimpinellifolium*	soil	2008	Ecuador/S00°41′27.1″/W90°19′21.9″/189 m	1-2	AB515362
EPp411-5rs	*S. pimpinellifolium*	soil	2008	Ecuador/S00°41′27.1″/W90°19′21.9″/189 m	1-2	AB515363
EPp411-8rs	*S. pimpinellifolium*	soil	2008	Ecuador/S00°41′27.1″/W90°19′21.9″/189 m	1-2	AB515364
EPp411-10rs	*S. pimpinellifolium*	soil	2008	Ecuador/S00°41′27.1″/W90°19′21.9″/189 m	1-1	AB515365
EPp411-13rs	*S. pimpinellifolium*	soil	2008	Ecuador/S00°41′27.1″/W90°19′21.9″/189 m	1-1	AB515366
EPp411-16s	*S. pimpinellifolium*	soil	2008	Ecuador/S00°41′27.1″/W90°19′21.9″/189 m	1-1	AB515367
EPp412s	*S. pimpinellifolium*	soil	2008	Ecuador/S00°41′27.1″/W90°19′21.9″/189 m	1-2	AB515368
EPp43-1s	*S. pimpinellifolium*	soil	2008	Ecuador/S00°41′23.0″/W90°19′10.3″/208 m	1-2	AB515369
EPp43-2s	*S. pimpinellifolium*	soil	2008	Ecuador/S00°41′23.0″/W90°19′10.3″/208 m	1-2	AB515370
EPp43-3s	*S. pimpinellifolium*	soil	2008	Ecuador/S00°41′23.0″/W90°19′10.3″/208 m	1-2	AB515371
EPp43-4s	*S. pimpinellifolium*	soil	2008	Ecuador/S00°41′23.0″/W90°19′10.3″/208 m	1-1	AB515372
EPp45-1s	*S. pimpinellifolium*	soil	2008	Ecuador/S00°40′05.2″/W90°16′08.9″/253 m	1-2	AB515373
EPp45-4s	*S. pimpinellifolium*	soil	2008	Ecuador/S00°40′05.2″/W90°16′08.9″/253 m	1-2	AB515374
EPp45-5s	*S. pimpinellifolium*	soil	2008	Ecuador/S00°40′05.2″/W90°16′08.9″/253 m	1-2	AB515375
EPp45-7s	*S. pimpinellifolium*	soil	2008	Ecuador/S00°40′05.2″/W90°16′08.9″/253 m	1-2	AB515376
PPp10-351s	*S. pimpinellifolium*	soil	2010	Peru/S12°05′0.70″/W76°56′32.2″/248 m	1-2	AB627138
PPp10-361s	*S. pimpinellifolium*	soil	2010	Peru/S12°04′34.8″/W76°56′31.1/247 m	1-2	AB627139
PPp10-36223s	*S. pimpinellifolium*	soil	2010	Peru/S12°04′34.8″/W76°56′31.1/247 m	1-1	AB627140
PPp11-5721s	*S. pimpinellifolium*	soil	2011	Peru/S11°19′15.7″/W76°52′19.0″/1269 m	1-2	AB697971
PPp11-801s	*S. pimpinellifolium*	soil	2011	Peru/S17°00′40.8″/W72°01′21.2″/139 m	1-1	AB697972
PPp11-802s	*S. pimpinellifolium*	soil	2011	Peru/S17°00′40.8″/W72°01′21.2″/139 m	1-1	AB697973
PPp11-8021s	*S. pimpinellifolium*	soil	2011	Peru/S17°00′40.8″/W72°01′21.2″/139 m	1-1	AB697974
PPp11-8022s	*S. pimpinellifolium*	soil	2011	Peru/S17°00′40.8″/W72°01′21.2″/139 m	1-1	AB697975
PPp11-811s	*S. pimpinellifolium*	soil	2011	Peru/S17°00′10.6″/W72°02′20.0″/132 m	1-1	AB697976
PPp11-8121s	*S. pimpinellifolium*	soil	2011	Peru/S17°00′10.6″/W72°02′20.0″/132 m	1-1	AB697977
PPp11-821s	*S. pimpinellifolium*	soil	2011	Peru/S17°00′20.0″/W72°02′18.7″/130 m	1-1	AB697978
PPp11-8221s	*S. pimpinellifolium*	soil	2011	Peru/S17°00′20.0″/W72°02′18.7″/130 m	1-2	AB697979
PPp11-8222s	*S. pimpinellifolium*	soil	2011	Peru/S17°00′20.0″/W72°02′18.7″/130 m	1-1	AB697980
***F. oxysporum*** **from transition tomatoes**						
MCE-77	*S. lycopersicum* var. *cerasiforme*	leaf	2005	Mexico/N20°13′08.6″/W98°39′14.6″/2281 m	nt	AB373873
MCE-9515s	*S. lycopersicum* var. *cerasiforme*	soil	2005	Mexico/N20°24′21.4″/W89°45′25.2″/40 m	1-1	AB373874
MCE-952s	*S. lycopersicum* var. *cerasiforme*	soil	2005	Mexico/N20°24′21.4″/W89°45′25.2″/40 m	1-2	AB373875
MCE10-C2s	*S. lycopersicum* var. *cerasiforme*	soil	2010	Mexico/N21°00′05.0″/W98°32′10.8″/638 m	1-1	AB627141
MCE10-C3s	*S. lycopersicum* var. *cerasiforme*	soil	2010	Mexico/N21°00′05.0″/W98°32′10.8″/638 m	1-1	AB627142
MCE10-C5s	*S. lycopersicum* var. *cerasiforme*	soil	2010	Mexico/N21°00′05.0″/W98°32′10.8″/638 m	1-1	AB627143
MCE10-E14s	*S. lycopersicum* var. *cerasiforme*	soil	2010	Mexico/N21°00′05.2″/W98°32′16.3″/635 m	1-1	AB627144
MCE10-E19s	*S. lycopersicum* var. *cerasiforme*	soil	2010	Mexico/N21°00′05.2″/W98°32′16.3″/635 m	1-2	AB627145
MCE10-F11s	*S. lycopersicum* var. *cerasiforme*	soil	2010	Mexico/N21°00′05.7″/W98°32′16.6″/655 m	1-1	AB627146
MCE10-F12s	*S. lycopersicum* var. *cerasiforme*	soil	2010	Mexico/N21°00′05.7″/W98°32′16.6″/655 m	1-1	AB627147
MCE10-F16s	*S. lycopersicum* var. *cerasiforme*	soil	2010	Mexico/N21°00′05.7″/W98°32′16.6″/655 m	1-1	AB627148
MCE10-F18s	*S. lycopersicum* var. *cerasiforme*	soil	2010	Mexico/N21°00′05.7″/W98°32′16.6″/655 m	1-1	AB627149
MCE10-J52s	*S. lycopersicum* var. *cerasiforme*	soil	2010	Mexico/N21°03′30.8″/W98°16′33.6″/449 m	1-1	AB627150
MCE10-J58s	*S. lycopersicum* var. *cerasiforme*	soil	2010	Mexico/N21°03′30.8″/W98°16′33.6″/449 m	1-2	AB627151
ME-2m	*S. lycopersicum* (jitomate criollo)	fruit	2005	—d	1-1	AB373881
ME-7s	*S. lycopersicum* (jitomate criollo)	soil	2005	Mexico/N17°24′22.4″/W92°02′01.0″/400 m	1-2	AB373889
ME-8s	*S. lycopersicum* (jitomate criollo)	soil	2005	Mexico/N17°24′22.4″/W92°02′01.0″/400 m	1-2	AB373882
ME-12s	*S. lycopersicum* (jitomate criollo)	soil	2005	Mexico/N17°24′22.4″/W92°02′01.0″/400 m	1-1	AB373883
ME-15s	*S. lycopersicum* (jitomate criollo)	soil	2005	Mexico/N17°24′22.4″/W92°02′01.0″/400 m	1-1	AB373887
ME-19s	*S. lycopersicum* (jitomate criollo)	soil	2005	Mexico/N17°24′22.4″/W92°02′01.0″/400 m	1-2	AB373884
ME-23s	*S. lycopersicum* (jitomate criollo)	soil	2005	Mexico/N17°24′22.4″/W92°02′01.0″/400 m	1-1	AB373885
ME-42s	*S. lycopersicum* (jitomate criollo)	soil	2005	Mexico/N17°24′22.4″/W92°02′01.0″/400 m	1-1	AB373888
ME-44s	*S. lycopersicum* (jitomate criollo)	soil	2005	Mexico/N17°24′22.4″/W92°02′01.0″/400 m	1-1	AB373886
ME9-6713s	*S. lycopersicum* (jitomate criollo)	soil	2009	Mexico/N20°03′08.5″/W97°33′13.7″/587 m	1-2	AB591425
ME9-67110s	*S. lycopersicum* (jitomate criollo)	soil	2009	Mexico/N20°03′08.5″/W97°33′13.7″/587 m	1-1	AB591426
ME9-67111s	*S. lycopersicum* (jitomate criollo)	soil	2009	Mexico/N20°03′08.5″/W97°33′13.7″/587 m	1-2	AB591427
ME9-6721s	*S. lycopersicum* (jitomate criollo)	soil	2009	Mexico/N20°03′08.5″/W97°33′13.7″/587 m	1-1	AB591428
ME9-67212s	*S. lycopersicum* (jitomate criollo)	soil	2009	Mexico/N20°03′08.5″/W97°33′13.7″/587 m	1-2	AB591429
*F****. oxysporum*** **from modern tomato cultivars**						
CE2-8DE	*S. lycopersicum*	fruit	2002	Chile/S18°29′29.7″/W70°16′19.5″/97 m	1-2	AB373839
CE2-17	*S. lycopersicum*	fruit	2002	Chile/S18°29′29.7″/W70°16′19.5″/97 m	1-1	AB373840
CE2-5	*S. lycopersicum*	stem	2002	Chile/S18°30′08.3″/W70°13′17.4″/184 m	1-2	AB373838
CE2-18	*S. lycopersicum*	stem	2002	Chile/S18°29′29.7″/W70°16′19.5″/97 m	1-1	AB373841
CE2-19	*S. lycopersicum*	stem	2002	Chile/S18°29′29.7″/W70°16′19.5″/97 m	1-2	AB373842
CE4-12	*S. lycopersicum*	stem	2004	Chile/S18°31′32.8″/W70°13′03.8″/213 m	1-2	AB373860
CE4-15	*S. lycopersicum*	stem	2004	Chile/S18°31′32.8″/W70°13′03.8″/213 m	1-1	AB373861
CE4-16	*S. lycopersicum*	stem	2004	Chile/S18°31′32.8″/W70°13′03.8″/213 m	1-2	AB373863
CE4-19	*S. lycopersicum*	stem	2004	Chile/S18°31′32.8″/W70°13′03.8″/213 m	1-2	AB373859
CE4-392	*S. lycopersicum*	stem	2004	Chile/S18°31′32.8″/W70°13′03.8″/213 m	1-1	AB373858
CE4-398s	*S. lycopersicum*	soil	2004	Chile/S18°31′32.8″/W70°13′03.8″/213 m	1-1	AB373866
CE4-3912s	*S. lycopersicum*	soil	2004	Chile/S18°31′32.8″/W70°13′03.8″/213 m	1-2	AB373867
CE4-3916s	*S. lycopersicum*	soil	2004	Chile/S18°31′32.8″/W70°13′03.8″/213 m	1-1	AB373868
CE4-3917s	*S. lycopersicum*	soil	2004	Chile/S18°31′32.8″/W70°13′03.8″/213 m	1-2	AB373864
CE4-3924s	*S. lycopersicum*	soil	2004	Chile/S18°31′32.8″/W70°13′03.8″/213 m	1-2	AB373870
CE4-3927s	*S. lycopersicum*	soil	2004	Chile/S18°31′32.8″/W70°13′03.8″/213 m	1-1	AB373865
ME-51	*S. lycopersicum*	leaf	2005	Mexico/N19°49′10.4″/W97°48′34.1″/1753m	1-2	AB373876
ME-54	*S. lycopersicum*	leaf	2005	Mexico/N19°49′10.4″/W97°48′34.1″/1753m	nt	AB373877
ME-53	*S. lycopersicum*	root	2005	Mexico/N19°49′10.4″/W97°48′34.1″/1753m	nt	AB373879
ME-55	*S. lycopersicum*	root	2005	Mexico/N18°47′13.9″/W99°10′29.7″/1184m	nt	AB373880
AMEs	*S. lycopersicum*	soil	2007	Afghanistan/N34°31′10.0″/E69°12′10.7″/1814 m	1-2	AB373936
ASEs	*S. lycopersicum*	soil	2007	Afghanistan/N34°49′26.7″/E69°15′05.6″/1591 m	1-2	AB373937
AGEs	*S. lycopersicum*	soil	2007	Afghanistan/N33°35′27.5″/E69°14′08.0″/2306 m	1-2	AB515352
ItE-1	*S. lycopersicum*	leaf	2007	Italy/N40°49′01.9″/E14°21′25.2″/148 m	1-2	AB373918
ItE-2s	*S. lycopersicum*	soil	2007	Italy/N40°49′04.9″/E14°22′18.1″/238 m	1-2	AB373919
ItE-3s	*S. lycopersicum*	soil	2007	Italy/N40°49′04.9″/E14°22′18.1″/238 m	1-2	AB373920
ItE-4s	*S. lycopersicum*	soil	2007	Italy/N40°49′04.9″/E14°22′18.1″/238 m	1-2	AB373922
ItE-5s	*S. lycopersicum*	soil	2007	Italy/N40°49′01.9″/E14°21′25.2″/148 m	1-1	AB373923
ItE-6s	*S. lycopersicum*	soil	2007	Italy/N40°49′01.9″/E14°21′25.2″/148 m	1-2	AB373924
ItE-8s	*S. lycopersicum*	soil	2007	Italy/N40°49′01.9″/E14°21′25.2″/148 m	1-1	AB373926
ItE-10s	*S. lycopersicum*	soil	2007	Italy/N40°49′01.9″/E14°21′25.2″/148 m	1-2	AB373927
ItE-11s	*S. lycopersicum*	soil	2007	Italy/N40°49′04.9″/E14°22′18.1″/238 m	1-2	AB373928
ItE-12s	*S. lycopersicum*	soil	2007	Italy/N40°49′01.9″/E14°21′25.2″/148 m	1-1	AB373929
ItE-14s	*S. lycopersicum*	soil	2007	Italy/N40°49′01.9″/E14°21′25.2″/148 m	1-2	AB373930
ItE-15s	*S. lycopersicum*	soil	2007	Italy/N40°49′01.9″/E14°21′25.2″/148 m	1-1	AB373921
ItE-16s	*S. lycopersicum*	soil	2007	Italy/N40°49′04.9″/E14°22′18.1″/238 m	1-2	AB373925
ItE-19s	*S. lycopersicum*	soil	2007	Italy/N40°49′01.9″/E14°21′25.2″/148 m	1-1	AB373931
ItE-21s	*S. lycopersicum*	soil	2007	Italy/N40°49′01.9″/E14°21′25.2″/148 m	1-2	AB373932
ItE-23s	*S. lycopersicum*	soil	2007	Italy/N40°49′01.9″/E14°21′25.2″/148 m	1-2	AB373933
ItE-29s	*S. lycopersicum*	soil	2007	Italy/N40°49′01.9″/E14°21′25.2″/148 m	1-2	AB373934
ItE-31s	*S. lycopersicum*	soil	2007	Italy/N40°49′04.9″/E14°22′18.1″/238 m	1-1	AB373935
JKE-15	*S. lycopersicum*	flower	2007	Japan/N32°52′06.1″/E130°33′12.3″/0 m	1-1	AB373894
JKE-1	*S. lycopersicum*	root	2007	Japan/N32°52′06.1″/E130°33′12.3″/0 m	1-1	AB373890
JKE-3	*S. lycopersicum*	root	2007	Japan/N32°52′06.1″/E130°33′12.3″/0 m	1-2	AB373891
JKE-5	*S. lycopersicum*	root	2007	Japan/N32°52′06.1″/E130°33′12.3″/0 m	1-2	AB373892
JKE-6	*S. lycopersicum*	root	2007	Japan/N32°52′06.1″/E130°33′12.3″/0 m	1-1	AB373893
JKE-11s	*S. lycopersicum*	soil	2007	Japan/N32°52′06.1″/E130°33′12.3″/0 m	1-2	AB373895
JKE-26s	*S. lycopersicum*	soil	2007	Japan/N32°52′06.1″/E130°33′12.3″/0 m	1-2	AB373896
JKE-27s	*S. lycopersicum*	soil	2007	Japan/N32°52′06.1″/E130°33′12.3″/0 m	1-1	AB373897
JKE-28s	*S. lycopersicum*	soil	2007	Japan/N32°52′06.1″/E130°33′12.3″/0 m	1-2	AB373899
JKE-29s	*S. lycopersicum*	soil	2007	Japan/N32°52′06.1″/E130°33′12.3″/0 m	1-2	AB373898
JKE-31s	*S. lycopersicum*	soil	2007	Japan/N32°52′06.1″/E130°33′12.3″/0 m	1-1	AB373900
JKE-34s	*S. lycopersicum*	soil	2007	Japan/N32°52′06.1″/E130°33′12.3″/0 m	1-2	AB373901
JIE-1s	*S. lycopersicum*	soil	2007	Japan/N36°21′19.0″/E136°22′15.4″/23 m	1-1	AB373902
JIE-2s	*S. lycopersicum*	soil	2007	Japan/N36°21′19.0″/E136°22′15.4″/23 m	1-1	AB373903
JIE-4s	*S. lycopersicum*	soil	2007	Japan/N36°21′19.0″/E136°22′15.4″/23 m	1-2	AB373905
JIE-7s	*S. lycopersicum*	soil	2007	Japan/N36°21′19.0″/E136°22′15.4″/23 m	1-2	AB373904
JIE-13s	*S. lycopersicum*	soil	2007	Japan/N36°21′19.0″/E136°22′15.4″/23 m	1-2	AB373906
JIE-15s	*S. lycopersicum*	soil	2007	Japan/N36°21′19.0″/E136°22′15.4″/23 m	1-1	AB373907
JIE-16s	*S. lycopersicum*	soil	2007	Japan/N36°21′19.0″/E136°22′15.4″/23 m	1-2	AB373908
JIE-17s	*S. lycopersicum*	soil	2007	Japan/N36°21′19.0″/E136°22′15.4″/23 m	1-2	AB373909
JIE-18s	*S. lycopersicum*	soil	2007	Japan/N36°21′19.0″/E136°22′15.4″/23 m	1-2	AB373910
JIE-19s	*S. lycopersicum*	soil	2007	Japan/N36°21′19.0″/E136°22′15.4″/23 m	1-2	AB373911
JIE-20s	*S. lycopersicum*	soil	2007	Japan/N36°21′19.0″/E136°22′15.4″/23 m	1-1	AB373912
JTE-1s	*S. lycopersicum*	soil	2007	Japan/N35°41′05.8″/E139°29′13.6″/62 m	1-1	AB373913
JTE-2s	*S. lycopersicum*	soil	2007	Japan/N35°41′05.8″/E139°29′13.6″/62 m	1-2	AB373914
JTE-3s	*S. lycopersicum*	soil	2007	Japan/N35°41′05.8″/E139°29′13.6″/62 m	1-1	AB373915
JTE-4s	*S. lycopersicum*	soil	2007	Japan/N35°41′05.8″/E139°29′13.6″/62 m	1-2	AB373916
JTE-5s	*S. lycopersicum*	soil	2007	Japan/N35°41′05.8″/E139°29′13.6″/62 m	1-1	AB373917

a N: North, S: South, W: West, E: East, lat/long is shown as dd°mm′ss.s″. (d: degree, m: minute, s: second).

b *S. peruvianum* in Mexico was cultivated for experimental purpose.

c Not tested.

d ME-2m was isolated from a jitomate criollo fruit sold in a Mexican market, latitude/longitude/altitude were not measured.

**Table 2:** Nucleotide primers used in this study

Name	Sequence (5′-3′)	Targeting gene/region	Thermal conditions	Amplicon size^a^	Reference
FIGS11	GTAAGCCGTCCTTCGCCTCG	ribsomal DNA IGS region	94°C 2 min; 30 × (94°C 1 min, 60°C 30 s, 72°C 1 min); 72°C 6 min	600 bp	(22)
FIGS12	GCAAAATTCAATAGTATGGC	ribsomal DNA IGS region			(22)

Gfmat1a	GCAAAATTCAATAGTATGGC	*MAT1-1-1* alpha-box (*MAT1-1*)	94°C 2 min; 30 × (94°C 30 s, 58°C 30 s, 72°C 45 s); 72°C 6 min	280 bp	(19)
Gfmat1b	TAAGCGCCCTCTTAACGCCTTC	*MAT1-1-1* alpha-box (*MAT1-1*)			(19)
GfHMG11	TACCGTAAGGAGCGTCAC	*MAT1-2-1* HMG-box (*MAT1-2*)		220 bp	(19)
GfHMG12	GTACTGTCGGCGATGTTC	*MAT1-2-1* HMG-box (*MAT1-2*)			(19)

P12-F2	GTATCCTCCGGATTTTGAGC	*SIX1* (*AVR3*)	94°C 2 min; 32 x (94°C 30 s, 58°C 45 s, 72°C 2 min); 72°C 7 min	840 bp	(41)
P12-R1	AATAGAGCCTGCAAAGCATG	*SIX1* (*AVR3*)			(51)
SIX3-F1	CCAGCCAGAAGGCCAGTTT	*SIX3* (*AVR2*)		570 bp	(51)
SIX3-R2	GGCAATTAACCACTCTGCC	*SIX3* (*AVR2*)			(51)
SIX4F	ACTCGTTGTTATTGCTTCGG	*SIX4* (*AVR1*)		800 bp	(19)
SIX4R	CGGAGTGAAGAAGAAGCTAA	*SIX4* (*AVR1*)			(19)

a Approximate size is shown.

**Table 3:** Previously described fungal strains used in this study

Fungal strain	Host plant^a^	Source^b^	Strain No.	Origin	Mating type	GenBank Accession No.^c^
*Fusarium oxysporum*						
f. sp. *lycopersici*	*Solanum lycopersicum*					
race 1		MAFF	103036	Japan	1-1	AB106020
		NBRC	6531	Japan	1-1	AB106018
		H. C. Kistler	OSU-451B	USA	1-1	AB106026
		NRRL	26034	Italy	1-1	AB106025
		M. Bon	CT-1	France	1-1	AB120970
		MAFF	103038	Japan	1-1	AB106031
race 2		MAFF	103043	Japan	1-1	AB106032
		JCM	12575	Japan	1-1	AB106027
		Y. Hirano	Saitama-ly2	Japan	1-1	AB373817 ^*^
		H. C. Kistler	MN-66	USA	1-1	AB106036
		A. D. Pietro	4287	Spain	1-1	AB120973
		R. Allende	mx-20	Mexico	1-1	AB373818 ^*^
race 3		Y. Hosobuchi	F-1-1	Japan	1-2	AB106037
		Y. Hosobuchi	H-1-4	Japan	1-2	AB106038
		T. Arie	tomino1-c	Japan	1-2	AB106044
		C. Yoshioka	Chz1-A	Japan	1-2	AB373819 ^*^
		H. C. Kistler	DA-1/7	USA	1-2	AB106047
		E. Vivoda	F240	USA	1-1	AB120976
		R. Allende	mx-4	Mexico	1-1	AB373820 ^*^
f. sp. *radicis-lycopersici*	*Solanum lycopersicum*	MAFF	103047	Japan	1-2	AB106059
		Y. Hirano	Saitama-rly	Japan	1-2	AB373821 ^*^
		Y. Hirano	Saitama-rly2	Japan	1-2	AB373822 ^*^
		A. Vermunt	NetRL	The Netherlands	1-1	AB373823 ^*^
		This study	CE-391s	Chile	1-1	AB373869 ^*^
f. sp. *melonis*	*Cucumis melo*	NRRL	26406	USA	1-2	AB106056
f. sp. *batatas*	*Ipomoea batatas*	MAFF	103070	Japan	1-2	AB106049
f. sp. *spinaciae*	*Spinacia oleracea*	T. Arie	880803e-2	Japan	1-2	AB373824
f. sp. *lactucae*	*Lactuca sativa*	T. Arie	SB1-1	Japan	1-2	AB373825 ^*^
f. sp. *asparagi*	*Asparagus officinalis*	F. Kodama	FokF233	Japan	1-2	AB373827 ^*^
f. sp. *conglutinans*	*Brassica oleracea* var. *capitata*	T. Yoshida	Cong: 1-1	Japan	1-1	AB106051
f. sp. *niveum*	*Citrullus lanatus*	MAFF	305608	Japan	1-2	AB106057
f. sp. *cucumerinum*	*Cucumis sativus*	T. Arie	Rif-1	Japan	1-1	AB106052
f. sp. *melongenae*	*Solanum melongena*	MAFF	103051	Japan	1-1	AB106055
f. sp. *apii*	*Cryptotaenia japonica*	SUF	1017	Japan	1-2	AB106048
f. sp. *matthioli*	*Matthiola incana*	T. Arie	880116a	Japan	1-1	AB106054
f. sp. *glycines*	*Glycine max*	T. Arie	851209m	Japan	1-1	AB373826 ^*^
f. sp. *fragariae*	*Fragaria* spp.	T. Arie	851209e	Japan	1-1	AB106053
nonpathogenic		Y. Amemiya	Fo304	Japan	1-1	AB373828 ^*^
		K. Watanabe	101-2	Japan	1-1	AB373829 ^*^
		S. Suwa	F4	Japan	1-1	AB373830 ^*^
		K. Watanabe	9901	Japan	1-1	AB373831 ^*^
		A. Vermunt	MDI31216059	The Netherlands	1-1	AB373832 ^*^
*Fusarium sacchari*	*Saccharum officinarum*	FGSC	7610	USA	1-2	GU170582

a Each host plant corresponds to formae specialis (f. sp.)

b MAFF, Microorganisms Section of the Gene Bank in the Ministry of Agriculture, Forestry and Fisheries of Japanese Government (Tsukuba, Ibaraki, Japan); NRRL, Agriculture Research Service Culture Collection of United State Department of Agriculture (Peolia, IL, USA); SUF, Culture Collection of Fusarium in Shinshu University (Ueda, Nagano, Japan); FGSC, Fungal Genetics Stock Center (University of Kansas Medical Center, Kansas city, KS, USA); CBS, Centraalbureau voor Schimmelcultures (Baarn, The Netherlands); NBRC, NITE (National Institute of Technology and Evaluation) Biological Resource Center (Kazusakamatari, Chiba, Japan); JCM, Japan Collection of Microorganisms (Tsukuba, Ibaraki, Japan)

c Asterisks show the sequence data registrated in this study.

**Table 4:** Susceptibility of wild and transition tomatoes (*Solanum* section *Lycopersicon*) to *F. oxysporum* f. sp. *lycopersici*

Sample Name	Sampled year	Sampled country	*F. oxysporum* f. sp. *lycopersici*^a^		

			race 1	race 2	race 3
*S. chilense*					
Lc0036	2002	Chile	0.0	0.0	0.0
*S. peruvianum*					
Lp0043-1	2004	Chile	0.0	0.0	0.0
Lp0044	2004	Chile	0.0	0.0	0.0
Lp0046	2004	Chile	0.0	0.0	0.0
*S. pimpinellifolium*					
Lpp0040	2008	Ecuador	1.0	0	1.0
Lpp0041w1	2008	Ecuador	1.0	1.0	1.0
Lpp0043	2008	Ecudoar	1.0	2.0	1.0
Lpp0045	2008	Ecuador	1.0	1.0	1.0
*S. lycopersicum* var. *cerasiforme*					
Lec0001	2005	Mexico	2.0	2.0	3.0
*S. lycopersicum* (jitomate criollo)					
Lecr0001	2005	Mexico	2.0	3.0	3.0
*S. lycopersicum*					
cv. Ponderosa (control)	—	—	2.0	1.0	2.0

a MAFF 305121, JCM 12575, and Chz1-A were used as race 1, 2, and 3 isolate for positive control. Inner symptom was estimated as follows. 0 (no symptoms) to 4 (death) scale.
