# Supplementary figures and images for: The Tomato Wilt Fungus Fusarium oxysporum f. sp. lycopersici shares Common Ancestors with Nonpathogenic F. oxysporum isolated from Wild Tomatoes in the Peruvian Andes
Source: Microbes Environ. 2014 Jun 6;29(2):200–10. doi: 10.1264/jsme2.ME13184 (PMC4103527; doi:10.1264/jsme2.ME13184)

Fig. S1

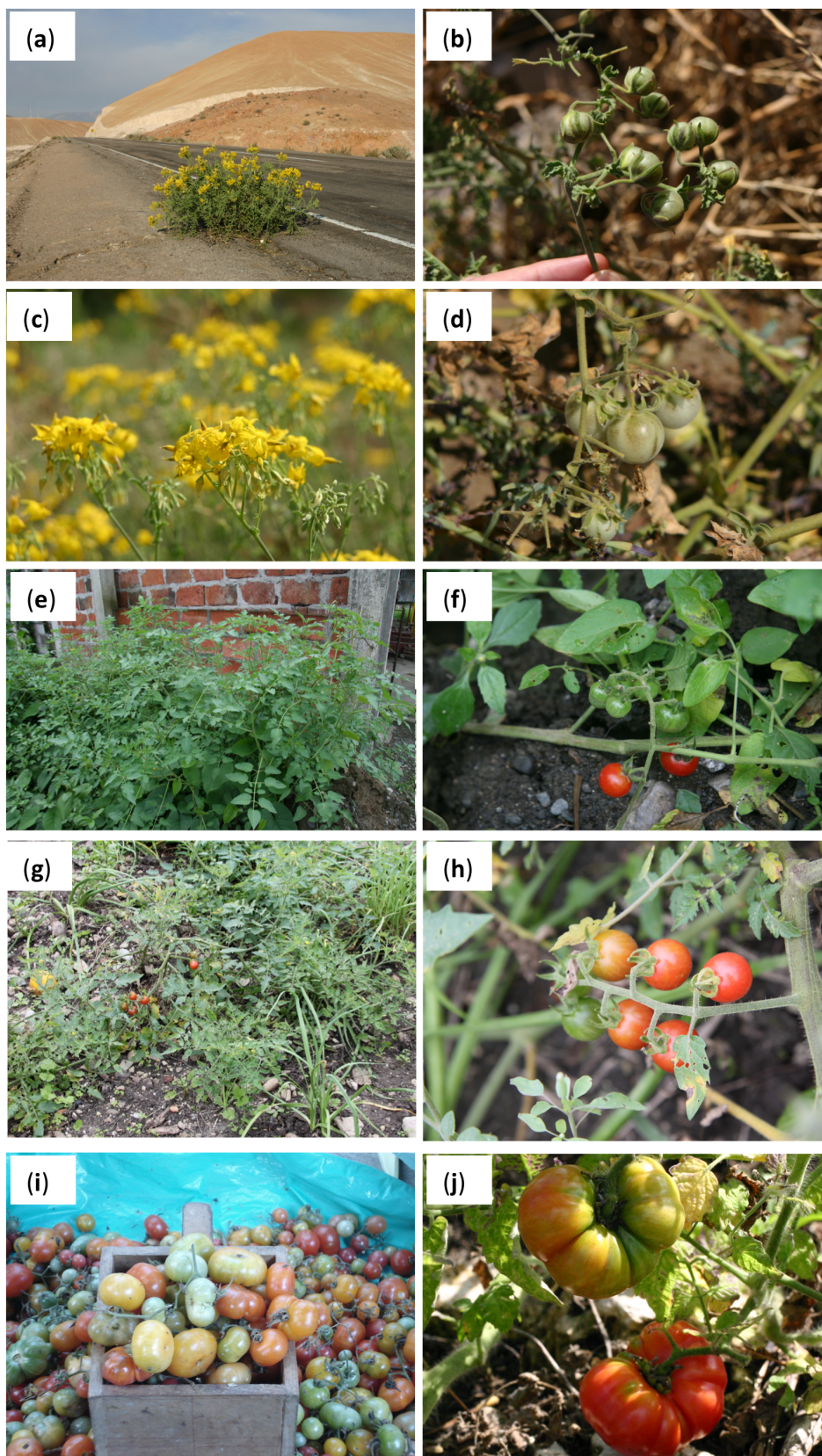

Supplement: Supplementary file 1 [file 29_200_s1.pdf]
